# Impact of Metabolic Syndrome Factors on Testosterone and SHBG in Type 2 Diabetes Mellitus and Metabolic Syndrome

**DOI:** 10.1155/2018/4926789

**Published:** 2018-07-02

**Authors:** Mukhtar Mohammed, Molham AL-Habori, Ahmed Abdullateef, Riyadh Saif-Ali

**Affiliations:** Department of Biochemistry and Molecular Biology, Faculty of Medicine and Health Sciences, University of Sana'a, Sana'a, Yemen

## Abstract

**Objective:**

Several studies have often reported low testosterone and SHBG to be associated with type 2 DM and the metabolic syndrome (MetS). Our objective was to determine the impact of metabolic syndrome and diabetic parameters on testosterone and SHBG in both MetS subjects and type 2 DM patients.

**Methods:**

In this study, 120 Yemeni male aged 30–70 years old were enrolled, 30 of whom were healthy subjects with BMI < 25 kg/m^2^ that served as control, 30 MetS, 30 type 2 DM without MetS, and 30 type 2 DM with MetS according to IDF criteria.

**Results:**

Testosterone (free and total) and SHBG were significantly lower in MetS subjects and modestly reduced in type 2 DM with and without MetS. Stepwise linear regression showed free and total testosterone to be negatively affected by waist circumference, and univariate analysis shows this significant difference to disappear when adjusted for waist circumference. On the other hand, stepwise linear regression showed SHBG to be positively affected by testosterone and age and negatively affected by FBG and TG. Univariate analysis shows this observed significant difference to disappear when adjusted for testosterone.

**Conclusion:**

Abdominal obesity is a major determinant of low testosterone levels irrespective of diabetes status. Thus, supporting evidence suggesting that the causative relationship between the often low testosterone and type 2 DM might be bidirectional or even multidirectional and interrelated with obesity, MetS, and IR.

## 1. Introduction

Type 2 diabetes mellitus (DM) is the predominant form of diabetes and accounts for at least 90% of all diagnosed cases. International diabetes federation estimates that 9% of the world's population (415 million) has diabetes in 2015, and this number is predicted to rise to 10% (642 million) by 2040, of whom 91% are diagnosed with type 2 DM [1]. It is a heterogeneous group of disorders that exhibit relative insulin deficiency and is usually associated with obesity, insulin resistance, impaired insulin secretion, and increased hepatic glucose production. Individuals with type 2 DM often show disturbances consistent with the metabolic syndrome (MetS) [2]. On the other hand, individuals with the MetS have increased risk of developing type 2 DM [3]. Metabolic syndrome presents the cluster of risk factors for atherosclerotic cardiovascular disease and type 2 DM that include raised blood pressure, dyslipidemia (raised triglycerides and lowered high-density lipoprotein cholesterol), raised fasting glucose, and central obesity [4], with insulin resistance being proposed as the key linking factor for the MetS diseases.

The association between late-onset hypogonadism and type 2 DM has been demonstrated in numerous studies [5, 6], indicating that up to 50% of men with MetS and insulin resistance states [5, 7] and up to 40% of men with type 2 DM have low testosterone levels, assessed as total, free, or bioavailable testosterone [8–10]. Several studies of hypogonadism in men with type 2 DM have inferred that low testosterone is an independent risk factor for type 2 DM, suggesting that testosterone supplementation therapy may be of benefit in some diabetic males including reduction of fasting glucose [11, 12]. Longitudinal population studies show that low testosterone is an independent risk factor for the development of both MetS and type 2 DM in later life [13–16] and their clinical sequels such as stroke or transient ischemic attacks [17]. Epidemiologic and genetic studies have also inferred a role for sex hormone-binding globulin (SHBG) in the pathogenesis of insulin resistance, MetS, and type 2 DM [18–20]. Low serum SHBG levels are associated with insulin resistance and hyperinsulinemia [18], suggesting that SHBG could be a new risk factor and predictor for the incidence of type 2 DM [21, 22]. Other studies have also shown that low testosterone and SHBG levels to also predict the development of MetS as well as diabetes [23] and that elevated testosterone and SHBG led to increased insulin sensitivity and reduced risk of MetS [24].

The contribution of the diabetic state per se relative to the effects of obesity and comorbidities to lowered testosterone in type 2 DM remains unclear with consequent implications for the therapeutic approach. We hypothesize that testosterone levels are related to the metabolic syndrome state and are not prevalent in type 2 DM. The present study compares the plasma levels of testosterone and SHBG between obese and nonobese type 2 DM versus nonobese normoglycemic controls, as well as determines the impact of metabolic syndrome factors and diabetic parameters on testosterone and SHBG in both MetS subjects and type 2 DM patients.

## 2. Methods

### 2.1. Subjects and Study Design

This case-control study was performed on 120 Yemeni male subjects aged 30–70 years who were recruited from the Endocrine Unit in Al-Thawra Hospital, Sana'a. Thirty were healthy subjects with body mass index (BMI) < 25 kg/m^2^ that served as controls, 30 subjects with metabolic syndrome (nondiabetic), 30 subjects with type 2 DM without metabolic syndrome, and 30 subjects with type 2 DM with metabolic syndrome according to IDF criteria. Subjects excluded include those with type 2 DM treated with insulin (since exogenous insulin will give false high insulin results), with hypogonadism or treated for hypogonadism (since those treated for hypogonadism will affect testosterone levels), as well as with liver and renal diseases. The study protocol was approved by the institutional review board (IRB) of the Faculty of Medicine and Health Sciences, Sana'a University. Informed consent was obtained from all individuals after explaining the purpose and nature of the study.

### 2.2. Demographic and Anthropometric Measurements

Body weight and height were measured, and BMI was computed as weight in kilograms (kg) divided by height in meters squared (m^2^). Waist circumference was measured midway between the lower rib margin and the superior iliac spine at the end of gentle expiration in a standing position. Blood pressure (BP) measurements were taken from each patient's right arm in the seated position by using an automatic blood pressure monitor after 10 min of rest in a quiet room. Two to three successive BP readings were obtained at 5-minute intervals and averaged.

### 2.3. Biochemical Analysis

Fasting venous blood samples (6 ml) were collected from each subject in the morning after 12-hour fast using Vacutainer plain tubes and separated by centrifugation within 10 minutes of collection at 3000 rpm for 10 minutes. The separated serum from each sample was divided into two separate Eppendorf tubes; one of them was stored at −20°C for testosterone, SHBG, and insulin measurements. Glucose and lipid profiles were determined immediately after sample collection.

Plasma glucose (FPG), triglyceride (TG), and HDL cholesterol (HDL-c) were measured by chemistry autoanalyzer (Siemens Healthcare Diagnostics Inc., USA). Testosterone, SHBG, and insulin were measured by electrochemiluminescence immunoassay (ECL) on cobas E-411 (Roche Diagnostics, Germany). Insulin resistance and *β*-cell function were calculated using the homeostasis model assessment (HOMA 2) calculator v2.2 which is available from Oxford Centre for Diabetes, Endocrinology and Metabolism. Free and total testosterone was calculated from serum total testosterone and SHBG using an online calculator (https://www.healthcare.siemens.com).

### 2.4. Statistical Analysis

The statistical analyses were performed on Social Package of Social Sciences (SPSS) version 11.5 (SPSS Inc., Chicago, IL, USA). ANOVA was used for comparing the means of parameters under study between normal subjects, metabolic syndrome, and type 2 DM with and without metabolic syndrome. The cofactors that affect testosterone, SHBG, HOMA-*β*, and HOMA-IR levels were screened with bivariate correlation (Pearson correlation), to be included in stepwise linear regression analysis. The stepwise linear regression was applied to assist the impact of metabolic syndrome factors and diabetic parameters on testosterone, SHBG, HOMA-*β*, and HOMA-IR. General linear model with univariate analysis (ANCOVA) was applied to compare testosterone, SHBG, HOMA-*β*, and HOMA-IR among different groups adjusted for cofactors confirmed by stepwise linear regression analysis. The accepted level of significance was set below 0.05 (*p* < 0.05).

## 3. Results

The general characteristics of the study population are listed in Table 1.  Table 2 elucidates the relationship between free and total testosterone and SHBG with the tested diabetic parameters and metabolic syndrome factors, whereby plasma total testosterone level was positively correlated with SHBG and negatively correlated with metabolic syndrome factors (BMI, waist circumference, SBP, and DBP) and diabetic parameters (insulin, HOMA-IR, and HOMA-*β*). However, free testosterone was only negatively correlated with BMI, waist circumference, SBP, and DBP, respectively. Plasma SHBG levels were negatively correlated with metabolic syndrome factors (BMI, waist circumference, and TG) and diabetic parameters (insulin and HOMA-IR).

Table 3 elucidates the impact factor of the metabolic syndrome factors and diabetic parameters on testosterone (free and total) and SHBG via stepwise linear regression. Controlling for the other parameters that showed significant correlation in Table 2, both free and total testosterone levels were negatively affected by waist circumference, whereas total testosterone was positively affected by SHBG. On the other hand, plasma SHBG levels were positively affected by age and testosterone and negatively affected by FBG and TG.

Table 4 shows a comparison of free and total testosterone and SHBG among different groups using ANOVA and general linear model with univariate analysis (ANCOVA). Plasma free and total testosterone and SHBG were significantly different (*p* = 0.01, *p* = 0.0002, and *p* = 0.009) in all tested groups as shown by ANOVA but were only significantly (*p* = 0.015, *p* = 8.6 × 10^−5^, and *p* = 0.004) lower in MetS by 22.4%, 32.8%, and 27.5% as compared to healthy normal subjects. SHBG was nonsignificantly lower in type 2 DM by 20% and that of total testosterone was nonsignificantly lower in type 2 DM with MetS by 16.4% with respect to the health normal subjects. On further usage of the general linear model with univariate analysis (ANCOVA) adjusting for waist circumference, free testosterone was not significantly different in all tested groups, whereas on adjusting for SHBG, total testosterone remained significantly (*p* = 0.017) lower in the MetS subjects as compared to the other groups. However, on comparing MetS and type 2 DM with and without MetS, the total testosterone was lower in MetS subjects (*p* = 0.059 and *p* = 0.006, resp.) (data not shown). On further adjustments for both SHBG and waist circumference, the significant difference previously observed in the total testosterone levels disappeared. Similarly, on adjusting for age, FBG, and TG, the level of SHBG remained significantly (*p* = 0.002) lower in the MetS group. However, on comparing MetS and type 2 DM with and without MetS, SHBG was lower in MetS subjects (*p* = 0.037 and *p* = 0.029, resp.) (data not shown). On further adjusting for testosterone, the significant difference in the SHBG levels disappeared.

## 4. Discussion

The impact of metabolic syndrome on free and total testosterone and SHBG was investigated in this study. Abdominal obesity was a major determinant of low testosterone levels irrespective of diabetes status. Both testosterone (free and total) and SHBG were significantly lower in MetS subjects, with a modest but not significant decrease in type 2 DM with and without MetS with respect to healthy normal subjects. These results are in line with other studies [25, 26]. Our results of the MetS subjects are in accordance with cross-sectional studies in which low levels of testosterone and SHBG have been associated with metabolic syndrome or its components including abdominal obesity, insulin resistance or hyperinsulinemia, dyslipidemia, and impaired glucose metabolism [27–29]. Numerous epidemiological studies over the past decades have shown a high prevalence of low testosterone levels in men with the MetS [5, 13] and confirmed the association of low total as well as calculated free testosterone levels in men with MetS compared with healthy control individuals. A recent study further suggested an association between low serum testosterone levels and MetS with a large proportion of the study patients having angiographically proven coronary artery disease [30]. Analyzing the data of 1139 US nationally representative group of men 20+ years old showed men with prediabetes to have lower serum total testosterone and SHBG than men without prediabetes [31].

Meta-analyses of case-control studies have reported type 2 DM to be associated with a modest but significant decrease in testosterone levels (around 3 nmol/l for total testosterone, and 10 pmol/l for free testosterone) [13, 32]. This discrepancy of the testosterone levels in our nonobese type 2 DM patients may be attributed to the fact that the majority of previous studies have assessed testosterone levels in obese type 2 DM patients [12, 13, 33, 34]. Several studies have also showed that obese men or those with insulin resistance tend to be androgen deficient [11, 35, 36]. Moreover, circulating SHBG was shown to be strongly associated with multiple circulating lipids and metabolites reflecting the degree of adiposity and insulin resistance in men [37] suggesting that low testosterone may be a marker of a metabolic imbalance affecting SHBG production in the liver. The question of causality is important as the answer has profound consequences for diagnosis, management, and prevention of these adverse health conditions.

Current evidence suggests that the causative relationship between the often low testosterone and type 2 DM might be bidirectional or even multidirectional and interrelated with obesity, MetS, SHBG, and other factors [11, 38]. The association of type 2 DM to low total testosterone was suggested to be secondary to the low SHBG level and thereby just reflects an adjustment of the pituitary gonadostat to a lower level to sustain the same level of free testosterone [39]. Low levels of serum SHBG are also frequently observed in states of insulin resistance-related conditions and have emerged as a predictor for the incidence of type 2 DM and metabolic syndrome [21, 25]. In obesity and hyperinsulinemia secondary to insulin resistance, there is a decrease in total testosterone related to lower SHBG levels resulting from either decreased hepatic synthesis of this protein [40] or a decrease in free testosterone to levels, which implies a real decline in testosterone production [9, 40, 41] in which Leydig cell steroidogenesis is impaired because of target organ resistance to insulin action and/or production of cytokines/hormones by the adipose tissue [35, 42]. Chronic perturbations in glucose metabolism and hyperinsulinemia have been shown to impair the Leydig cell steroidogenesis via insulin-mediated induction of DAX1 (dosage-sensitive sex reversal, adrenal hypoplasia critical region, on chromosome X, gene 1), thus causing testosterone deficiency in mice [43].

The results presented in this study also showed testosterone to be negatively correlated with insulin, HOMA-IR, and HOMA-*β*, which is in line with a number of studies [8, 44, 45]. Both free and total testosterone levels were negatively correlated with metabolic syndrome factors (BMI, waist circumference, SBP, and DBP), which is in agreement with several studies reporting a close and inverse association between low testosterone and individual cardiovascular risk factors such as obesity, insulin resistance, hypertension, and dyslipidemia and also indicating that testosterone therapy could improve glycemic control and dyslipidemia [12, 32, 34, 46]. Moreover, it is suggested that measures aimed at reducing adiposity and hyperglycemia in MetS and type 2 DM may be beneficial in their management and may improve their sexual function and enhance good quality of life [47]. A recent study reported that, apart from an inverse correlation between free testosterone and subcutaneous adipose tissue (SAT) cell size in univariate analyses, an inverse association of free testosterone levels with TG and HOMA-IR was observed [26], suggesting a direct negative impact of adipocytes, but not IR, on free and total testosterone levels in obese men.

On using stepwise linear regression and controlling for the parameters that previously showed significant correlation, waist circumference was an independent factor that decreases free and total testosterone levels, whereas SHBG increases total testosterone. The general linear model with univariate analysis (ANCOVA) showed that on adjustment for SHBG, testosterone remained lower in the MetS subjects as compared to the other groups. However, on further adjustments for both SHBG and waist circumference, the significant difference disappeared, thus suggesting a role for abdominal obesity in the association of testosterone with both MetS and type 2 DM. This is in line with the cross-sectional survey from the National Health and Nutrition Examination Survey (NHANES) III [48] reporting the difference of testosterone between type 2 DM and nondiabetic controls to be no longer significant after adjusting for BMI and waist to hip ratio (WHR). This is consistent with a bidirectional relationship between visceral fat and testosterone, whereby increased visceral fat leads to increased secretion of proinflammatory cytokines, estradiol, insulin, and leptin, all of which may inhibit the activity of the hypothalamopituitary gonadal axis at multiple levels [14, 15]. Low testosterone thus promotes further accumulation of visceral fat, which, via increased inflammatory cytokines, increases insulin resistance and diabetes. This complex pathophysiological interplay is termed the hypogonadal-obesity-adipocytokine hypothesis, which describes the bidirectional relationship between low levels of testosterone and the metabolic syndrome [5, 13, 49].

Moreover, in accordance with several reports [50, 51], our study also observed negative correlation of SHBG with metabolic syndrome factors (BMI, waist circumference, and TG) and diabetic parameters (insulin and HOMA-IR), which is in line with previous studies [8, 18, 44, 45]. Hyperinsulinemia and insulin resistance are associated with low SHBG levels [18], suggesting that insulin level and/or insulin resistance is suppressive to SHBG production. This concept is supported by earlier *in vitro* evidence in which insulin reduces SHBG secretion directly and also inhibits the stimulatory action of thyroxine and estradiol on its synthesis in human HepG2 cells [52, 53]. However, subsequent studies have found no direct association between SHBG and insulin or insulin resistance [54]. Moreover, an *in vitro* study has shown that SHBG is not regulated by insulin but rather repressed by monosaccharide-induced lipogenesis in human HepG2 cells [55], suggesting that fatty liver or liver dysfunction which is usually associated with insulin resistance and diabetes might be a mechanism for the lower SHBG level. Recently, insulin per se was demonstrated not to directly suppress SHBG *in vivo*, but rather the improvement of insulin resistance elevates SHBG after intensive insulin hypoglycemic therapy [51]. Using stepwise linear regression and controlling for the parameters that previously showed significant correlation, plasma SHBG levels remained affected positively by age and testosterone and negatively by FBG and TG. The univariate analysis adjustment for age, FBG, and TG showed that the level of SHBG remained lower in the MetS subjects as compared to other groups. However, on further adjusting for testosterone, the significant difference disappears.

On comparing MetS and type 2 DM with or without MetS, the improved (higher) levels of testosterone and SHBG observed in type 2 DM with and without MetS may be the consequence of the diabetic treatment that improves the insulin sensitivity and insulin concentration and subsequently improves testosterone and SHBG levels. An earlier study in nondiabetic men has shown that SHBG is negatively correlated to insulin secretory pulse frequency [56], thus further emphasizing the relationship between insulin sensitivity and circulating SHBG. This hypothesis is consistent with the increase in serum testosterone and SHBG concentrations observed with maneuvers such as weight loss through calorie restriction which enhance insulin sensitivity [11, 57, 58]. Losing 10% of the starting body weight leads to a rise in total testosterone of approximately 2–4 nmol/l, whereas bariatric surgery may even increase levels of total testosterone by up to 10 nmol/l [59]. Bariatric surgery was also reported to normalize testosterone, SHBG, BMI, TG, and HOMAIR as well as restore sexual function and fertility [26]. Androgen deprivation therapy for prostate cancer increases obesity, decreases insulin sensitivity, and may be associated with a greater incidence of diabetes [60]. Rapid lowering of circulating testosterone, either by ending testosterone replacement therapy in hypogonadal men or by experimentally blocking the action of endogenous testosterone in normal men, is quickly followed by elevations in fasting glucose [61].

### 4.1. Limitations

Our study is based on a single measurement of hormones and SHBG and lack information on symptoms of hypogonadism and/or gonadotropin levels. Hormonal and SHBG measurements were also conducted using immunoassay, which is less reliable and less specific than more recent methods such as the liquid chromatography tandem mass spectrometry that is used to determine steroidal hormone levels.

### 4.2. Conclusion

In conclusion, this case-control study demonstrates that abdominal adiposity is a major determinant of low testosterone, irrespective of diabetes, by examining the impact of metabolic syndrome factors and diabetic parameters on testosterone and SHBG using stepwise linear regression and confirmed by ANCOVA. Our study, unlike many previous studies, examined the testosterone and SHBG levels in both nonobese and obese type 2 DM patients and showed type 2 DM patients to be associated with a modest but not significant decrease in testosterone with respect to healthy normal subjects. This raises the question whether testosterone therapy decreases insulin resistance independent of visceral fat reduction.

## Figures and Tables

**Table 1:** Characterization of subjects included in the study.

	Normal (*n* = 30)	Metabolic syndrome (*n* = 30)	Type 2 DM without metabolic syndrome (*n* = 30)	Type 2 DM with metabolic syndrome (*n* = 30)	ANOVA *p* value
Age (years)	38 ± 7.7	44 ± 8.5	47 ± 9.7	51 ± 5.7	**0.001**
BMI	22.6 ± 2.3	30.2 ± 2.9	25.3 ± 2.5	28.1 ± 2.4	**1.4 × 10** ^**−21**^
Waist circumference	85 ± 5.8	104 ± 6.7	91 ± 6.4	102 ± 5.4	**3.1 × 10** ^**−25**^
Systolic blood pressure	117 ± 9	136 ± 14	119 ± 15	136 ± 15	**5.9 × 10** ^**−9**^
Diastolic blood Pressure	76 ± 7	90 ± 10	77 ± 12	89 ± 12	**2.2 × 10** ^**−8**^
Triglyceride (mg/dl)	139 ± 51	199 ± 74	211 ± 81	233 ± 81	**1.8 × 10** ^**−5**^
HDL-c (mg/dl)	44 ± 8.6	42 ± 6.0	40 ± 5.7	40 ± 5.1	**0.039847**
FBG (mmol/l)	5.1 ± 0.4	5.5 ± 0.6	10.0 ± 3.7	10.7 ± 3.1	**6.2 × 10** ^**−19**^
Insulin (pmol/l)	50 ± 21	116 ± 35	72 ± 30	88 ± 34	**1.4 × 10** ^**−12**^
HOMA-IR	1.1 (0.9–1.2)	2.5 (2.2–2.8)	2.0 (1.6–2.4)	2.3 (2.0–2.7)	**1.6 × 10** ^**−12**^
HOMA-*β*	89 (79–102)	144 (128–162)	39 (31–49)	39 (31–49)	**1.5 × 10** ^**−21**^

**Table 2:** Correlation of free and total testosterone and SHBG with metabolic syndrome factors and diabetic parameters.

	Free testosterone	Total testosterone	SHBG
Testosterone	0.82 **(<0.001)**	—	—
SHBG	0.09 (0.33)	−0.63 **(<0.001)**	—
Age	−0.16 (0.08)	−0.05 (0.595)	0.14 (0.129)
BMI	−0.3 **(0.001)**	−0.43 **(<0.001)**	−0.37 **(<0.001)**
Waist circumference	−0.32 **(<0.001)**	−0.40 **(<0.001)**	−0.27 **(0.003)**
Systolic blood pressure	−0.24 **(0.008)**	−0.25 **(0.005)**	−0.11 (0.242)
Diastolic blood pressure	−0.21 **(0.02)**	−0.26 **(0.004)**	−0.14 (0.121)
TG	0.02 (0.85)	−0.09 (0.306)	−0.19 **(0.036)**
HDL-c	−0.05 (0.56)	−0.03 (0.752)	0.03 (0.710)
FBS	0.05 (0.62)	−0.04 (0.641)	−0.14 (0.139)
Insulin	−0.18 (0.049)	−0.32 **(<0.001)**	−0.30 **(<0.001)**
HOMA-IR	−0.17 (0.07)	−0.30 **(<0.001)**	−0.29 **(<0.001)**
HOMA-*β*	−0.16 (0.09)	−0.19 **(0.041**)	−0.10 (0.281)

The data are presented as *R*^2^ and *p* value.

**Table 3:** Impact of metabolic syndrome factors on free and total testosterone and SHBG by stepwise linear regression.

Impact of metabolic syndrome factors on free testosterone	
Waist circumference	−0.42 **(0.0003)**

Impact of metabolic syndrome factors on total testosterone	
SHBG	0.085 **(1.6 × 10**^**−12**^**)**
Waist circumference	−0.05 **(0.0009)**

Impact of metabolic syndrome factors on SHBG	
Age	0.38 **(0.0002)**
FBG	−0.044 **(0.01)**
TG	−0.024 **(0.028)**

The results are presented as *b* value and (*p* value).

**Table 4:** Comparing of free and total testosterone and SHBG among different groups using ANOVA and general linear model with univariate analysis (ANCOVA).

ANOVA								
					*p* value			
	Normal	MetS	T2DM without MetS	T2DM with MetS	ANOVA	MetS against normal	T2DM without MetS against normal	T2DM with MetS against normal

Testosterone (ng/ml)	6.1 (5.5-6.8)	4.1 (3.7–4.7)	5.4 (4.8–6.2)	5.1 (4.4-5.9)	**0.0002**	**8.6 × 10** ^**−5**^	0.5	0.15
SHBG (nmol/l)	40 (36–44)	29 (25–33)	32 (27–38)	33 (29–38)	**0.009**	**0.004**	0.11	0.2
Free testosterone (pmol/l)	420 (384–457)	326 (281–372)	415 (365–468)	383 (338–430)	**0.01**	**0.015**	0.999	0.66

ANCOVA								
					ANCOVA			

Free testosterone®	361 (310–419)	348 (303–400)	377 (334–425)	400 (352–454)	0.35	0.77	0.60	0.37
Testosterone^#^	5.8 (5.3–6.4)	4.8 (4.2–5.3)	5.8 (5.3–6.3)	5.5 (5.0–6.0)	**0.017**	**0.005**	0.84	0.28
Testosterone^&^	5.4 (4.8–6.1)	5.1 (4.5–5.8)	5.6 (5.1–6.1)	5.8 (5.2–6.3)	0.372	0.58	0.67	0.544
SHBG^$^	40 (34–46)	28 (24–33)	37 (32–41)	37 (32–43)	**0.002**	**0.0003**	0.41	0.56
SHBG^∗^	38 (33–42)	34 (30–38)	35 (31–38)	36 (32–41)	0.46	0.15	0.39	0.70

The results are presented as means and 95% confidence interval; ®general linear model adjusted for waist; ^#^general linear model adjusted for SHBG; ^&^general linear model adjusted for SHBG and waist; ^$^general linear model adjusted for FBG, TG, and age; ^∗^general linear model adjusted for FBG, TG, age, and testosterone.
